# Cook County Hospital

**Published:** 1877-09

**Authors:** 

**Affiliations:** House Physician


### Clinical Reports.

### COOK COUNTY HOSPITAL.

Services of Dr. H. A. Johnson.

(Reported by Dr. Park, House Physician.)

*Lead Poisoning.*

Case I. Lizzie C., aged 21, domestic. Admitted March 5th, 1877.

For three years she was in the habit of using the cosmetic known as "flake white," being ignorant of its deleterious properties. Has never been very robust, though enjoying ordinary good health in spite of attacks of scarlatina, typhoid fever, and trivial troubles, along with backache, leucorrhœa, and other disorders peculiar to her sex. One year ago, while suffering from typhoid fever, during the early stages of which she was salivated, she noticed sudden pain in hands and arms, followed by inability to use the hands. Recovery from the fever was not followed by return of manual power, though at times there has been partial improvement.

On admission she is evidently laboring under some cachexia; bowels are costive, tongue with a heavy white coat, appetite very poor. There is the characteristic line along the gums, marked fetor of breath, and paralysis of the extensors of both hands. There is considerable gastric irritability, with a feeling of oppression over hypogastrium; her menses have been absent for four months.

The treatment at first consisted of magnesium sulphate ℥ss, sulphuric acid dil. f℥ss, ter in die, with anodynes and carminatives as needed. Since her admission the treatment has been changed to nitro-muriatic acid, and again to potassic iodide. There has been a gradual amelioration of the symptons; the menstrual flow is now regular; the other excretory functions performed with ease and regularity. She has gained in flesh and spirits. The only vestiges of her former trouble are a faint line about the gums, and the drooping hands, which she has not yet the power to raise. The treatment to-day (Aug. 5th,) consists of tonics, sulphur baths and the induced current.

Case II. Susie A., a cyprian, aged 19. Admitted August 1st, 1877.

The history of this case is very similiar to that detailed above, the cosmetic used being the same, the period of its use only one year, and the method of applying it being to mix the powder with glycerine. The onset of the symptons was also similar, lassitude, weakness of the limbs, pain in the same, obstinate constipation, and paralysis of the extensors of the hand.

On admission; she is poorly nourished, skin sallow and clammy, functions of alimentary canal deranged, limbs almost useless, drooping hands, blue line about the gums, pulse 126 and small.

She was given a general tonic treatment of strychnia, mineral acids and bitter tonics, and the sulphate of magnesium as an eliminant.

Within a week she has manifested some improvement, but the time has been too short to warrant anything but a favorable prognosis.

*Mercurial Erethism.*

Henry B., native of Belgium, aged 18. Admitted June 21st, 1877.

For six months patient has been employed in a lookingglass factory, constantly handling the amalgam and exposed to its noxious influence. About six weeks ago he began to have severe headache, and his muscles to become very tremulous. Soon after appeared a profuse papular eruption over the whole surface of his body. His gums then became tender and mastication difficult. On admission; is well nourished, appetite and sleep good, tongue clean and moist, bowels regular, urine normal, skin natural. He complains of pain in the head; and the muscles of the extremities are in a peculiarly tremulous condition whenever he exerts them, and they have lost some of their tone as shown by the use of the dynamometer. The breath is not fetid, there is no salivary flow, and the gums only slightly altered in appearance.

He was given a sulphur bath every day, potassic iodide as an eliminant, and laxatives and anodynes as needed.

He made a rapid improvement as far as the muscular tremors were concerned, but as these symptoms wore away he began to show symptoms of hysteria, and slight, and finally acute, mania. Now (on the 7th of August,) he appears as stout and vigorous as could be desired; his muscles are firm, there are no tremors, the gums are no longer sore, the appetite is good, and he is the picture of health. It is only seldom now that he gives way to the crying fits and delusions which so troubled him two weeks ago.

